# Power law relationship between cell cycle duration and cell volume in the early embryonic development of *Caenorhabditis elegans*

**DOI:** 10.3389/fphys.2014.00529

**Published:** 2015-01-28

**Authors:** Yukinobu Arata, Hiroaki Takagi, Yasushi Sako, Hitoshi Sawa

**Affiliations:** ^1^Laboratory for Cell Fate Decision, Center for Developmental Biology, RIKENHyogo, Japan; ^2^Cellular Informatics Laboratory, RIKENSaitama, Japan; ^3^Department of Physics, School of Medicine, Nara Medical UniversityNara, Japan; ^4^Multicellular Organization Laboratory, National Institute of GeneticsShizuoka, Japan

**Keywords:** cell size, cell cycle duration, power law, nuclear-cytoplasmic volume ratio, *ima-3*/*Importin α*

## Abstract

Cell size is a critical factor for cell cycle regulation. In *Xenopus* embryos after midblastula transition (MBT), the cell cycle duration elongates in a power law relationship with the cell radius squared. This correlation has been explained by the model that cell surface area is a candidate to determine cell cycle duration. However, it remains unknown whether this second power law is conserved in other animal embryos. Here, we found that the relationship between cell cycle duration and cell size in *Caenorhabditis elegans* embryos exhibited a power law distribution. Interestingly, the powers of the time-size relationship could be grouped into at least three classes: highly size-correlated, moderately size-correlated, and potentially a size-non-correlated class according to *C. elegans* founder cell lineages (1.2, 0.81, and <0.39 in radius, respectively). Thus, the power law relationship is conserved in *Xenopus* and *C. elegans*, while the absolute powers in *C. elegans* were different from that in *Xenopus*. Furthermore, we found that the volume ratio between the nucleus and cell exhibited a power law relationship in the size-correlated classes. The power of the volume relationship was closest to that of the time-size relationship in the highly size-correlated class. This correlation raised the possibility that the time-size relationship, at least in the highly size-correlated class, is explained by the volume ratio of nuclear size and cell size. Thus, our quantitative measurements shed a light on the possibility that early embryonic *C. elegans* cell cycle duration is coordinated with cell size as a result of geometric constraints between intracellular structures.

## Introduction

Cell cycle is regulated in coordination with cell size in unicellular organisms and cells in animal embryos. In many invertebrate and vertebrate animals, the early embryonic stage after fertilization is characterized by rapid synchronous cleavage in all cells within the embryo. Later, this pattern of cell division abruptly changes to cycles of slow and asynchronous cleavage. This transition event was referred as the midblastula transition (MBT) originally in amphibian embryos (Gerhart, 1980; Newport and Kirschner, 1982). Experimental studies showed that the onset of events at or after MBT, such as asynchronous division, differentiation, and gastrulation, are affected by cell size as well as ploidy in *Drosophila* (Edgar et al., 1986) and *Xenopus* (Newport and Kirschner, 1982; Clute and Masui, 1995). These findings suggest that cell size and genome size are critical factors for determining the timing of MBT, which is the classic concept to explain the coordination between cellular events and cell size in early development of animal embryos.

Some variations of the classic concept have been reported based on quantitative measurements of cellular variables. Yoshio Masui and Wang reported that the cell cycle duration after MBT is inversely proportional to the cell radius squared in *Xenopus* embryos (Masui and Wang, 1998; Wang et al., 2000). Their rationale for this second power law relationship was that mitosis-promoting factor (MPF) is produced in a quantity proportional to the cell surface area. This hypothesis implies that the cell cycle durations coordinate with cell size through cell surface area, rather than volume.

On the other hand, other researchers proposed that the volume ratio between the cell and nucleus, but not the ploidy, directs the timing of blastomere adhesiveness in starfish and sea urchin embryos (Masui and Kominami, 2001; Masui et al., 2001). In starfish embryos, cell adhesiveness begins to increase after the eighth cleavage to form a monolayered hollow blastula. In accordance with the classic concept, the timing of adhesiveness was accelerated in embryos with doubled ploidy, whereas the timing was delayed in large-sized embryos by the fusion of a non-nucleate egg fragment. In contrast to the classic concept, the timing of adhesiveness was not altered in half-sized embryos, and the timing was only delayed by one cell cycle in quarter-sized embryos. They noticed that experimental manipulations changing cytoplasmic volume or changing ploidy altered the nuclear size, and they found that the cell adhesiveness appeared at a certain volume ratio of the nucleus to the cell (Masui et al., 2001). The same conclusion was derived from experimental observations of sea urchin embryos (Masui and Kominami, 2001). They concluded that the critical variable for determining the onset of blastomere adhesiveness in starfish and sea urchin embryos is the volume ratio between the nucleus and cell.

Thus, cellular events could be coordinated with cell size by the various ratios of cellular variables. However, quantitative measurements to reveal how cell cycle duration is coordinated with cell size have not been performed in embryos other than in the vertebrate, *Xenopus*. In the present work, we studied the time-size relationship in embryos of an invertebrate, *C. elegans*. In *C. elegans* embryo, the cell lineages and order of cell divisions are nearly invariant (Sulston et al., 1983; Schnabel et al., 1997). After fertilization, the P0 zygote divides into the large AB and smaller P1 daughters. Through several rounds of asymmetric cell division, the zygote eventually produces six founder cells: AB, MS, E, C, D, and P4.

Here, we report the time-size relationship—specifically, the cell cycle duration–cell volume (T–V) relationship—follows a power law relationship in *C. elegans*. Interestingly, the absolute powers differed among cell lineages in *C. elegans* and were less than the power in *Xenopus*. We discuss the possibility that cell cycle duration is coordinated with cell size through the volume ratio between nucleus and cell in *C. elegans* embryos. In addition, we discuss the difference and possible similarity of time-size relationships between *C. elegans* and *Xenopus* embryos.

## Materials and methods

### *C. elegans* culture conditions and recording of embryonic cell divisions

Wild-type *C. elegans* (N2) embryos were maintained at 22.5°C (Brenner, 1974). Embryos were isolated from gravid hermaphrodites. Cell divisions were recorded in a temperature-controlled room on an upright differential interference contrast (DIC) microscope with the Plan-Apochromat 63×/1.40 oil DIC objective lens (Carl Zeiss, Germany). Cell divisions were recorded at one-minute time intervals and 0.5-μm Z-axis intervals after the one-cell stage with Metamorph software (Molecular Devices, USA). Embryos were attached to a cover glass coated with polylysine (Sigma-Aldrich, USA). Cover glass was footed with petroleum jelly (Vaseline, Nacalai Tesque, Japan) on the slide glass. In this set-up, embryos that attached to the cover glass were separated from the slide glass, such that cell divisions proceed without the physical stress of compression between the cover glass and slide glass (Lee and Goldstein, 2003; Arata et al., 2010; Edgar and Goldstein, 2012). Polylysine attachment did not change the embryo shape (Figure S1). *C. elegans* embryos exhibited normal developmental progression and hatched in this setting.

### Measurements of cell volume

Cell volumes were measured by integrating 10 or more cylinder volumes (integral approach; IA). The volume of each cylinder was calculated from the cell area at each Z-plane and a constant height, which was set as the Z-axis interval in the stage control system of the Metamorph software (Molecular Devices, USA). The cell area in a cylinder was determined as shown in Figure S1A. The length of one pixel in the DIC image was calibrated by an objective micrometer (Carl Zeiss, Germany). Measurement error was estimated by comparing the nuclear volume measured by the IA to the volume measured by the formula approach (FA). In the FA, the nucleus in each cell was assumed to be a perfect sphere and its volume was determined by 4π*r*^3^ /3, by using the mean of four times measurements of the nuclear radius. The assumption is approximately correct, because the shape of the nucleus was close to a perfect circle in the X-Y and X-Z axes (Figure S1B). The measurement error in the IA was estimated to be 23.6% larger than the precise cell volume (Table 1). The error might be caused by integration error or slight elongation of the nucleus in the Z-axis (Figure S1B), probably due to the difference of refractive indices in the light path (Born and Wolf, 1999). Final cell volumes were determined by correcting measurement errors and averaging the volumes measured at three different time points during the cell cycle due to an absence of detectable cell growth in the embryonic cell cycle.

**Table 1:** **Volume correction measured by differential interference contrast (DIC) microscopy**.

**Cell identity**	**Radius ± SD (μm)**	**Volume by FA ± SD (μm^3^)**	**Volume by IA, no error correction (μm^3^)**	**Error (%)**
AB(1)	4.811 ± 0.150	467.4 ± 42.7	517.4	10.7
P(1)	4.436 ± 0.209	367.4 ± 52.1	433.7	18.0
AB(2)	4.321 ± 0.066	338.2 ± 15.7	433.0	28.0
AB(3)	3.845 ± 0.058	238.2 ± 10.7	312.4	31.2
AB(4)	3.132 ± 0.094	128.9 ± 11.3	164.7	27.7
AB(5)	2.871 ± 0.117	99.5 ± 12.2	116.2	16.8
AB(6)	2.352 ± 0.081	54.6 ± 5.7	72.7	33.0
Average ± SEM				23.6 ± 3.2

*The measurement error of the integral approach (IA) for our DIC microscope settings was estimated by comparing the nuclear volumes measured by the IA and by the formula approach (FA). The numbers in parentheses indicate the cell generation in each founder cell lineage. For example, AB(1) indicates the AB cell, and AB(2) indicates the AB daughter cells. See details in the Materials and Methods. SD, standard deviation; SEM, standard error of the mean*.

### Statistical analyses

To examine the power of the T–V relationship in cell lineages, the cell cycle duration and cell volume variables in the logarithmic or linear scale were fitted by the linear least-squares method or the Levenberg-Marquardt algorithm, respectively. To estimate the confidence interval (CI) of the estimated powers, a method combining regression analysis and a bootstrap method was used (Efron and Tibshirani, 1993). The values of power were resampled 10,000 times using residuals between experimental data and values derived from a model function. The 95% CIs were determined at the 2.5th and 97.5th percentiles in the appearance frequency of the values of power in linear scale data fitted by the Levenberg-Marquardt algorithm. The 95% CIs in the logarithmic scale fitted by the linear least-squares method were determined practically by the same method. The percentile method to estimate CIs can be applied to any symmetric statistical distribution.

## Results

### Quantitative determination of the T–V relationship in *C. elegans* embryos

**Figure 1:**
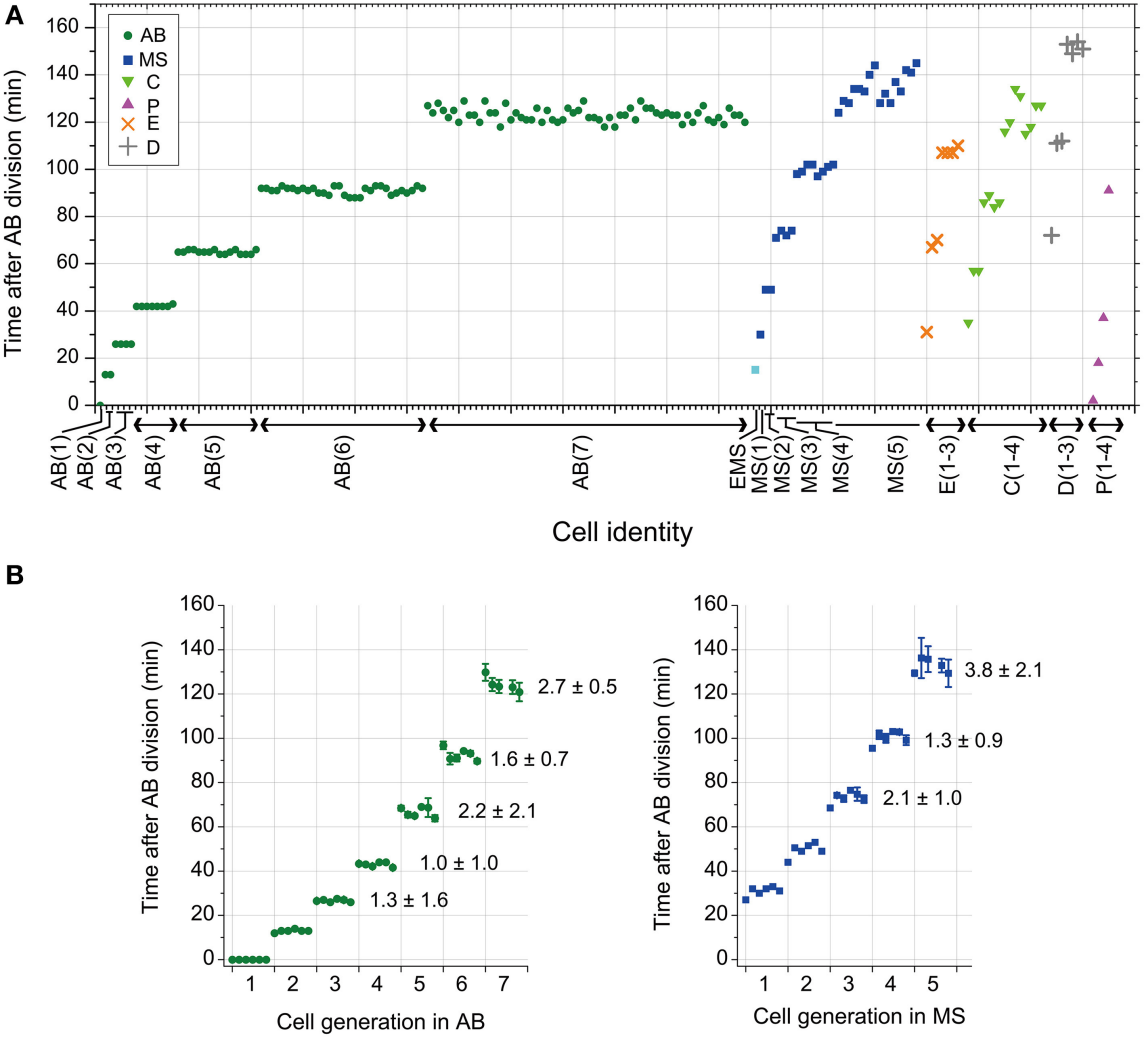
**Cell division timing of *C. elegans* embryos**. **(A)** The cell division timings in an embryo cultured at 25°C. Cell identity is indicated on the horizontal axis. Cell division timing was determined by nuclear envelope breakdown (NEBD). The numbers in parentheses indicate the cell generation in each founder cell lineage. For example, AB(1) indicates the AB cell, and AB(2) indicates the AB daughter cells. **(B)** The average and standard deviation (SD) of cell division timings in the same generation in AB and MS lineages in an embryo were obtained; data obtained from six embryos were aligned on the horizontal axis in order (the leftmost of AB and in the leftmost of MS were obtained from an embryo). The CV of cell division timings in the same generation in AB and MS lineages in an embryo were averaged among the six embryos and were shown with SD [the average CV ± SD (%)] in the right side of data in the graphs after the third generation. The NEBD of the AB cells was set as time 0. AB, MS, C, P, E, and D are indicated with a green dot, blue square, light green triangle, magenta triangle, orange x-mark, and gray cross, respectively. The EMS cell was indicated by a light blue square.

We observed the timing of cell division in wild-type *C. elegans* embryos cultured at 25°C. The intervals of cell divisions between the generations in the same lineage appeared to increase gradually in all the founder cell lineages in an embryo (Figure 1A). The average and standard deviation of coefficient of variation (CV) of cell division timings in AB and MS lineages among different embryos were around several percent (1 to 4 ± 0.5 to 2.1) (Figure 1B), indicating that the cell divisions occurred synchronously in a highly reproducible manner among embryos. Thus, the *C. elegans* embryo is a good model system to study a deterministic mechanism to regulate cell division timings in animal embryos.

**Figure 2:**
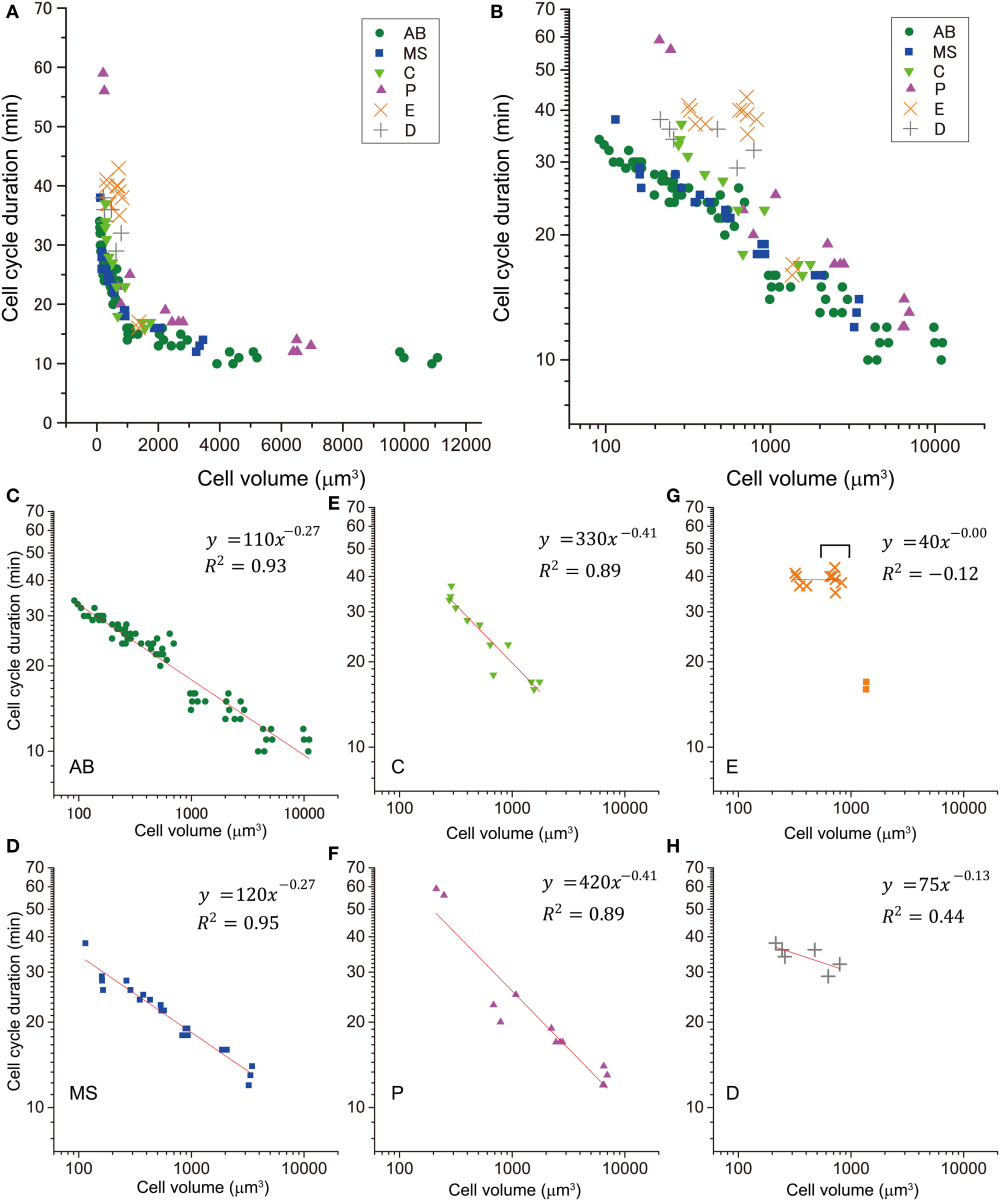
**Relationship between cell cycle duration and cell volume**. This relationship of cells in embryos is shown in a double logarithmic plot **(A)** and a linear plot **(B)**. The relationship of cells in AB [green dot, **(C)**], MS [blue square, **(D)**], C [light green triangle, **(E)**], P [magenta triangle, **(F)**], E [orange x-mark, **(G)**], and D [gray cross, **(H)**] lineages are shown in the double logarithmic plot. Cell volume and cell cycle duration data were obtained from four wild-type embryos. Data in the logarithmic scale were fitted to the formula, *y* = *a* + *bx*, by the linear least-squares method. **(G)** E cells are indicated by squares, and their descendants are indicated by orange x-marks. Downward bracket indicates the daughter cells of E cells. Regression analysis of cells in E lineage was performed without the E cells. Degrees of freedom in fitting in **(C–H)** were 68, 19, 10, 11, 8, and 4, respectively.

Next, we examined the T–V relationship. Cell cycle duration was defined as the time from nuclear formation in a cell to nuclear formation in one of the daughter cells, in which the nucleus was formed earlier. Cell cycle duration correlated negatively with cell volume (Figures 2A,B). When we classified the T–V relationship data by cell lineage, cell cycle duration vs. cell volume appeared linear in double logarithmic plots (Figures 2C–H), suggesting a power law relationship. We fitted three different models (Gaussian, exponential, and power law) to the plots of cell cycle duration vs. cell volume in linear scale. The χ^2^-value in the model fitting was smallest (except for the E lineage) when the data were fitted by the power law model (Figure S2). Therefore, we concluded that the *C. elegans* T–V relationship in the AB, MS, C, and P lineages follows a power law relationship.

**Figure 3:**
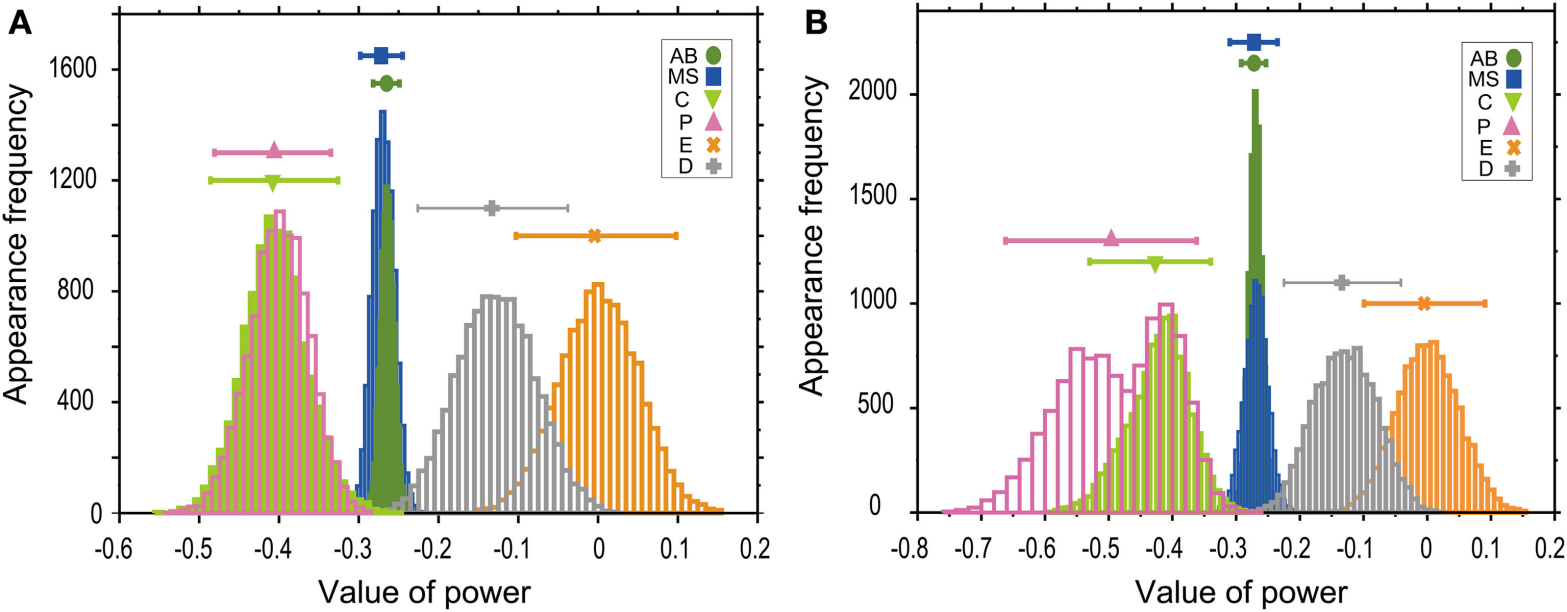
**Powers of the T–V relationship could be classified into three classes**. T–V relationships in each lineage in the logarithmic **(A)** or linear **(B)** scale were fitted by a power law model. Statistical analysis combining regression analysis and a bootstrap method were performed 10,000 times, using the same data used in Figure 2. The estimated power is indicated in the horizontal axis, while the appearance frequencies of the values of power is indicated in the vertical axis. The 95% CIs of the power of the T–V relationship were determined by the appearance frequency and are shown by long horizontal bars. Data for AB, MS, C, P, E, and D lineages are shown in green, blue, light green, magenta, orange, and gray, respectively.

Absolute values of power in the T–V relationship (Figure 2) were similar between AB and MS lineages (0.27) and between C and P lineages (0.41). Bootstrapping statistical analyses showed that the 95% CIs of the powers overlapped between AB and MS, C and P, and E and D lineages (Figures 3A,B). The larger absolute values of power in the C and P lineages indicated that the cell cycle duration elongates rapidly as the cell volume decreases (the highly size-correlated class). In contrast, the smaller absolute values of power in the AB and MS lineages indicated that the cell cycle duration elongates slowly (the moderately size-correlated class). When the power is zero, the cell cycle duration is constant or does not correlate with changes in cell size, indicating a size-non-correlated class. Cells in the E and D lineages exhibited lower values of power. Although it remains unclear due to small sample number, cells in the E and D lineages may be classified in another class with lower values of power, possibly the size-non-correlated class. These results suggest that the powers of the T–V relationship could be grouped into at least three classes.

In the bootstrap analysis performed to evaluate the T–V relationship in the logarithmic scale, the appearance frequencies of the values of power were symmetrically distributed (Figure 3A), which supports the validity of our estimation of the CIs. In the bootstrap analysis of the T–V relationship in the linear scale, the appearance frequencies of the AB, MS, E, and D lineages were symmetrically distributed, whereas the appearance frequencies of the C and P lineages showed monomodal and bimodal distributions with the shorter tail in the side of the larger values of power, respectively (Figure 3B). In these asymmetric distributions, the estimation of the CIs could be biased to the shorter tail side of the distributions. Because the similar skewness of the distributions was observed both in the C and P lineages, the asymmetry of the distributions does not affect our conclusion that the 95% CIs of the values of powers overlapped between the C and P lineages.

### Intermitotic phase duration elongates exponentially as cell volume decreases in the size-correlated classes

**Figure 4:**
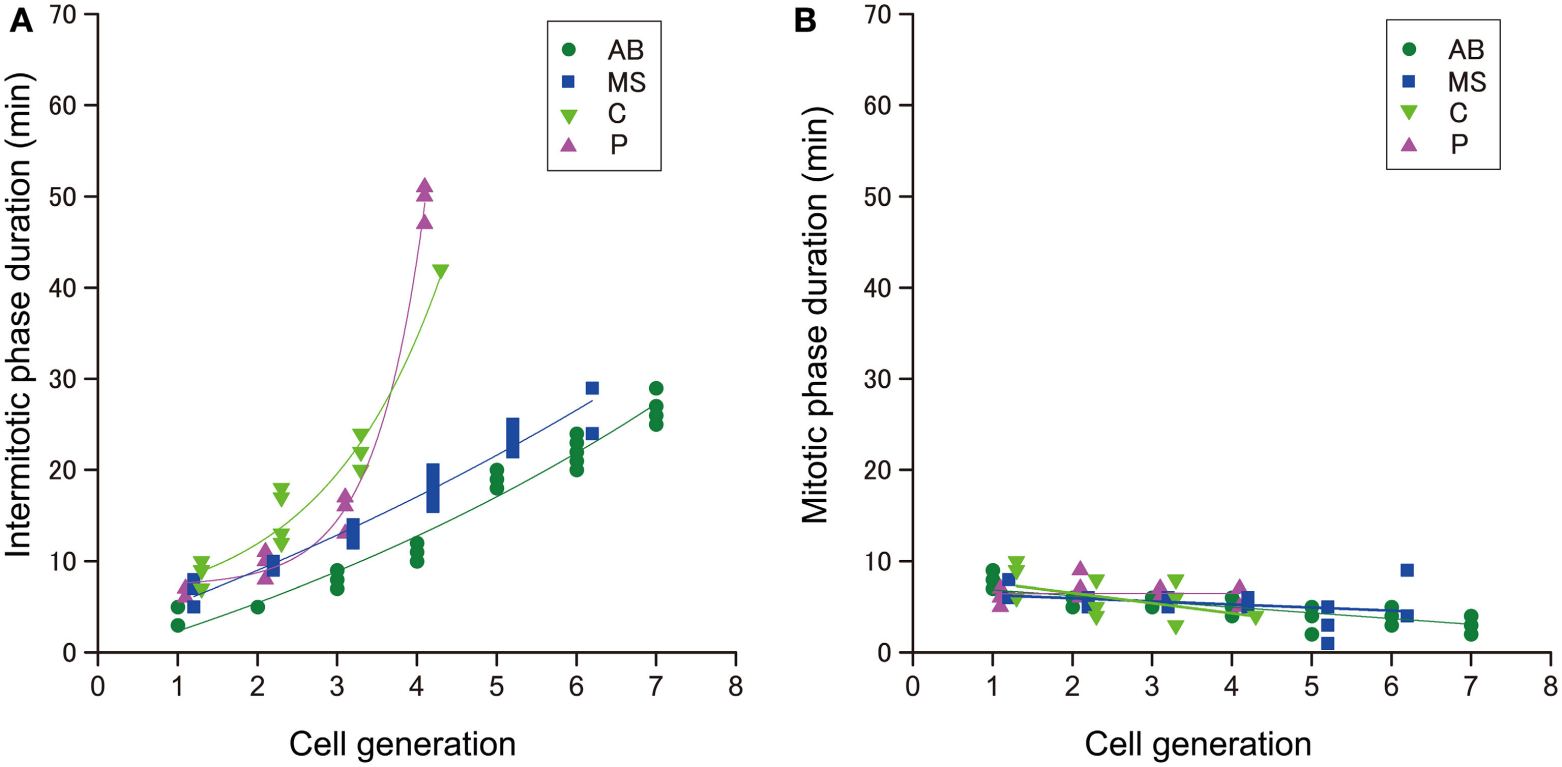
**The duration of cell cycle phases in *C. elegans* embryos**. The duration of the **(A)** intermitotic phase or **(B)** mitotic phase in the size-correlated class; AB (green dot), MS (blue square), C (light green triangle), and P (magenta triangle) lineages are shown in linear plots in the vertical axis. The cell generations in each founder cell lineage are shown in the horizontal axis. Data points are displaced along the horizontal axis to avoid overlap **(A,B)**. This data displacement does not affect exponentiation of data. Duration data were obtained from three wild-type embryos.

To determine which cell cycle phase was responsible for elongation of the cell cycle duration, we measured the duration of the intermitotic and mitotic phases in cells in the size-correlated AB, MS, C, and P lineages. The duration of the intermitotic phase was elongated exponentially as the rounds of cell division increased, and became dominant in cell cycle duration in later generations (Figure 4A), whereas the duration of the mitotic phase was relatively constant among these lineages (Figure 4B). These observations indicated that cell cycle elongation was due to lengthening of the intermitotic phase but not the mitotic phase.

### The relationship between the nuclear and cell volumes in the size-correlated classes

**Figure 5:**
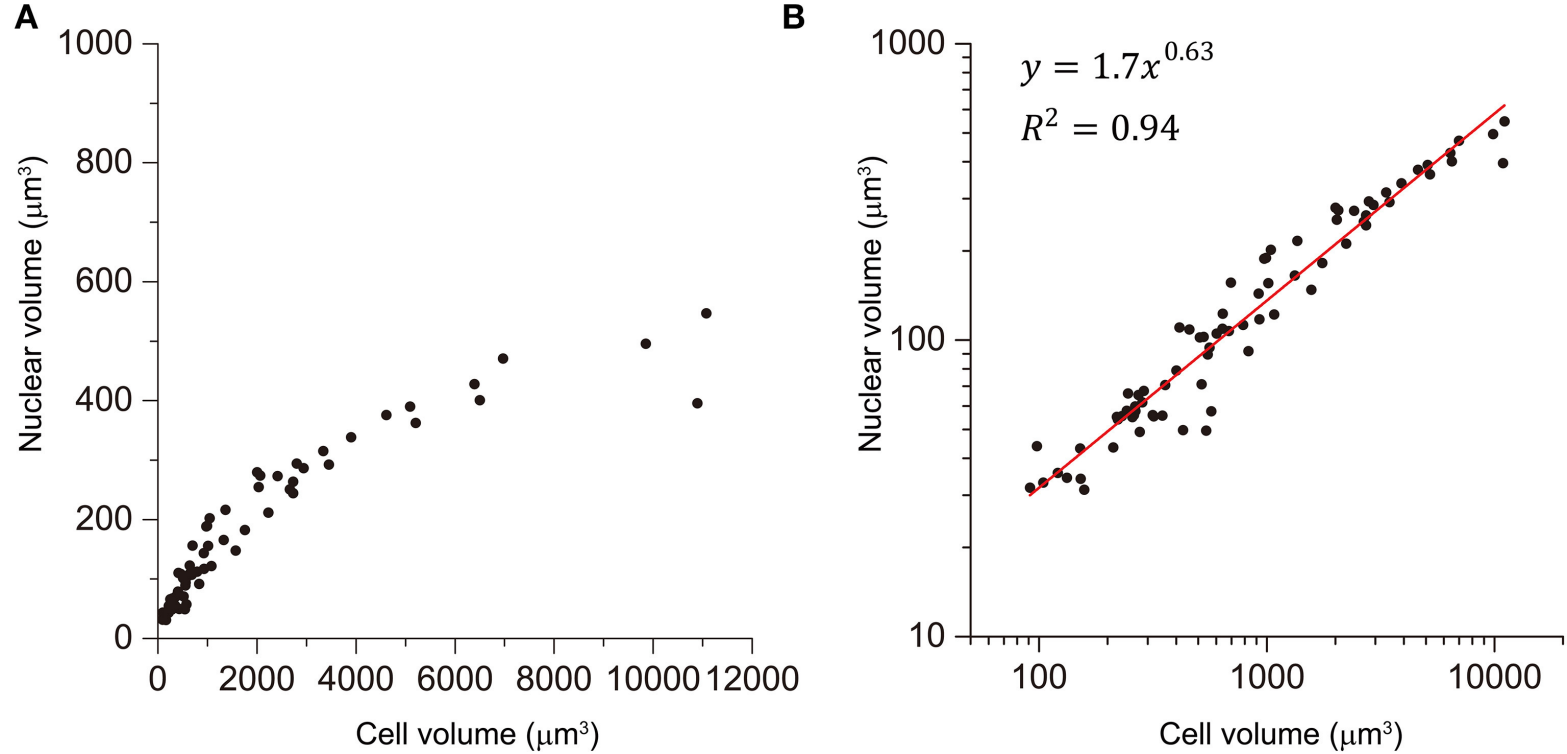
**Power law relationship between the nuclear and cell volume**. Relationship between nuclear and cell volume in size-correlated classes (AB, MS, C, and P) is shown in linear **(A)** and double logarithmic **(B)** plots. Cell volumes were determined by the integral approach with error correction, whereas nuclear volumes were determined by the formula approach in three wild-type embryos. Data in logarithmic scale were fitted to the formula, *y* = *a* + *bx*, by the linear least-squares method. Degree of freedom in fitting was 75.

To explain the *C. elegans* power law T–V relationship, we focused on the relationship between the cell and nuclear volumes. We plotted the nuclear vs. cell volumes for cells in size-correlated AB, MS, C, and P lineages (Figure 5A). The relationship between the nuclear and cell volumes was non-linear in a linear plot, and showed a linear relationship in a double logarithmic plot (Figure 5B). The relationship was well-fitted by a power law model (*R*^2^ = 0.94; Figure 5B). Nuclear volume varied with cell volume, in a power law relationship with a slope of 0.63 (Figure 5B). If the volumes of the two spheres varied in a corresponding manner, then the power was unity; thus, the *C. elegans* relationship between the nuclear and cell volumes was allometric. Supposing that a factor critical for cell cycle regulation is transported between the nucleus and cytoplasm, we considered the ratio of the nuclear volume (*V_n_*) to the cell volume (*V_c_*). The power of the volume ratio was −0.37 (*V_n_* /*V_c_* ∝ *V*^0.63^_*c*_ /*V_c_* = *V*^−0.37^_*c*_). We found that the absolute value of the power of the volume ratio (0.37) was closest to that of the T–V relationship in the highly size-correlated class (C and P lineages) (0.41), indicating a strong correlation with the volume ratio between the nucleus and cell.

### Genetic analysis of the T–V relationship in *C. elegans* embryos

**Figure 6:**
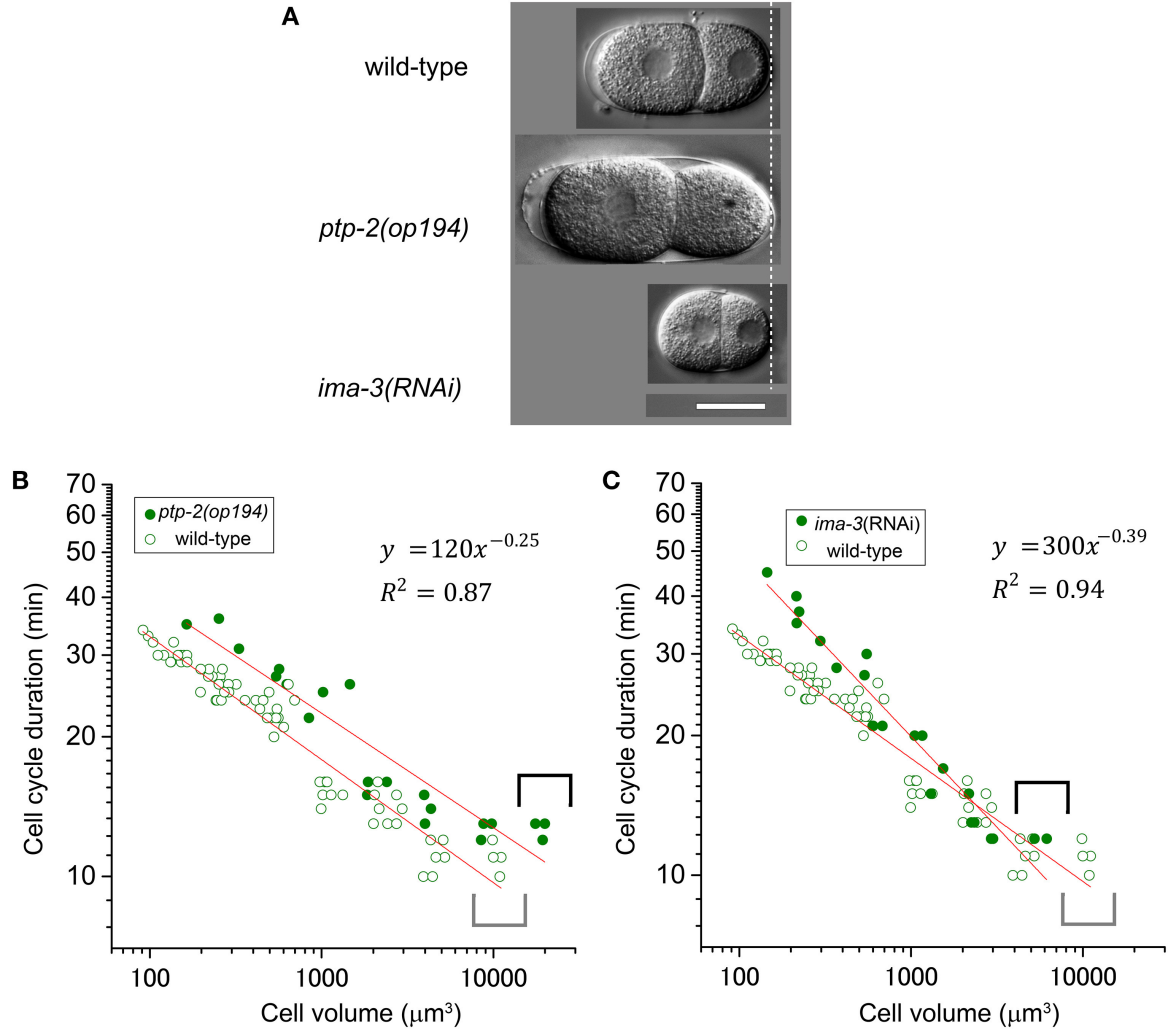
**Relationship between cell cycle duration and cell volume in loss-of-function embryos**. **(A)** Images of embryos at the two-cell stage for wild-type, *ptp-2(op194)*, and *ima-3* RNAi embryos were obtained by differential interference contrast (DIC) microscopy. Scale bar = 20 μm. Relationship between cell cycle duration and cell volume in AB lineage in *ptp-2(op194)* [filled circles in **(B)**] and *ima-3*(RNAi) [filled circles in **(C)**] embryos are shown with that in wild-type embryos [open circles in **(B,C)**] in double logarithmic plots. Cell volume and cell cycle duration data in loss-of-function embryos were obtained from each of three embryos. Data in the logarithmic scale were fitted to the formula, *y* = *a* + *bx*, by the linear least-squares method. Degrees of freedom in fitting **(B,C)** were 19 and 19, respectively.

We employed a genetic approach to assess the impact of altered cell volume and to examine the molecular mechanism of the T–V relationship. In *C. elegans*, genome-wide screening and classic genetics have identified genes related to egg size determination. Homozygous mutant embryos of *ptp-2*/SH2 domain-containing protein tyrosine phosphatase are larger than wild-type embryos, whereas *ima-3/importin* α RNAi embryos are smaller than wild-type embryos (Figure 6A) (Gutch et al., 1998; Sonnichsen et al., 2005). We measured cell cycle duration and cell volume in the AB lineage of these two loss-of-function embryos. Volumes of AB cells in the *ptp-2* mutant embryos and *ima-3* RNAi embryos were approximately twice and half the sizes, respectively, of AB cells from wild-type embryos (compare gray and black brackets in Figures 6B,C). The T–V relationship of the *ptp-2* AB lineage was well-fitted with a power law model with the absolute power, 0.25 (*R*^2^ = 0.87; Figure 6B), which was close to that of the wild-type AB lineage (0.27; Figure 2). Larger AB cells in *ptp-2* mutant embryos (black brackets) did not further shorten cell cycle durations compared to AB cells in wild-type embryos (gray brackets; Figure 6B). Thus, the cell cycle duration may have a minimum limit, and eventually appeared to have the minimum limit in the T–V relationship.

The power law relationship was maintained in the *ima-3* AB lineage, but the absolute value of the power of the *ima-3* RNAi embryos was increased (0.39, *R*^2^ = 0.94; Figure 6C) close to the absolute value of the power of the highly size-correlated class (C and P lineages) in wild-type embryos (0.41; Figure 2). Thus, *ima-3* is required to determine the proper cell cycle elongation, and eventually appeared to determine the proper power of the T–V relationship. The order of cell divisions in the *ima-3* RNAi embryos was the same as in the wild-type embryos, at least until the 16-cell stage (data not shown). Thus, it is unlikely that the rapid elongation of cell cycle in *ima-3* RNAi embryos was caused by defects in cell fate determination of the founder cells.

## Discussion

Cell cycle duration is coordinated with cell size in cultured mammalian cells and unicellular organisms. For example, *Amoeba proteus* cells did not enter the mitotic phase when the cell volume was reduced by cytoplasmic amputation (Prescott, 1955). By changing cell size with genetic or culture manipulations, cell cycle progression has been shown to be affected in budding yeast (Johnston et al., 1977), fission yeast (Nurse, 1975; Sveiczer et al., 1996), ciliates (Berger, 1984), and mammalian cultured cells (Dolznig et al., 2004). A quantitative relationship between cell cycle duration and cell size was reported in embryos of the vertebrate, *Xenopus* (Masui and Wang, 1998; Wang et al., 2000). In this work, we studied the quantitative relationship in embryos of an invertebrate, *C. elegans*.

### Powers of the T–V relationship among cell lineages in *C. elegans*

We found that the relationship between cell volume and cell cycle duration followed a power law relationship. In *C. elegans*, AB and P1 cells divide asynchronously, with the larger AB cells dividing before the smaller P1 cells (Sulston et al., 1983; Brauchle et al., 2003). The zygotes that are depleted of subunits of the heterotrimeric G-proteins, GOA-1/GPA-16, or GoLoco-containing proteins, GPR-1/GPR-2, exhibit normal anterior-posterior polarity, but divide into equally sized AB and P1 cells (Gotta and Ahringer, 2001; Colombo et al., 2003; Gotta et al., 2003; Srinivasan et al., 2003). The equally sized AB and P1 cells divide in a more synchronized manner compared to the differently sized AB and P1 cells in wild-type embryos. Thus, the cell cycle duration in AB and P1 cells is strongly correlated with cell size. Brauchle et al. proposed that unequal cell size of AB and P1 cells contributes to asynchrony of cell division, although it is still possible that G protein signaling is specifically required for differential checkpoint activation at the two-cell stage or for another cellular process modulating cell cycle progression (Brauchle et al., 2003). The power law relationship between cell cycle duration and cell size in later embryonic development may be regulated by a mechanism similar to the asynchrony of AB and P1 divisions.

Our statistical analyses suggest that there are at least three different classes of the T–V relationship according to the founder cell lineages in *C. elegans*: highly size-correlated, moderately size-correlated, and probably size-non-correlated classes (absolute powers of 0.41, 0.27, and <0.13 for volume, respectively). *C. elegans* founder cells give rise to different types of differentiated cells: cells in AB and MS lineages primarily produce ectodermal and mesodermal cells, C and P lineages produce mesodermal and germline cells, and E and D lineages produce endodermal and mesodermal cells, respectively (Sulston et al., 1983). Thus, the cell fates do not correlate with the classes according to the power of the T–V relationship. Instead, the classification might correlate with a mode of cell divisions. Cells in AB and MS lineages of the moderately size-correlated class share prominently synchronous and symmetric cell divisions (Figure 1) (Sulston et al., 1983), while cells in the P lineage undergo asymmetric cell divisions, in which the two daughter cells clearly differ in size from each other (Sulston et al., 1983). Similarly, many cells in the C lineage undergo asymmetric cell division, which has a clear size asymmetry (Figure S3). Thus, cells in P and C lineages in the highly size-correlated class share size-asymmetric cell division. It remains unclear what cell feature the E and D lineages share. Daughter cells of each E cell exhibited an abrupt deviation from the T–V relationship that the mother E cell followed, and subsequently, the descendants appeared to exhibit a lower power in the T–V relationship (Figures 2B,G). The E daughters are the first cells that have the Gap phase (G2 phase) in *C. elegans* embryos (Edgar and McGhee, 1988). Therefore, the deviation may be caused by the introduction of the Gap phase. This cell cycle dynamics in E lineage may be shared with D lineage.

Overall, our quantitative measurements revealed the diversity of powers in the T–V relationship among *C. elegans* cell lineages. Cells may sense their own size through distinct mechanisms among cell lineages in *C. elegans* embryos.

### Powers of the T–V relationship in *C. elegans* and *Xenopus*

We found that any of the absolute powers of the *C. elegans* lineages (<0.39, 0.81, and 1.2 in radius; <0.13, 0.27, and 0.41 in volume, respectively) were smaller than that in *Xenopus* embryos (2.0 in radius; 0.67 in volume). The cell cycle duration of *C. elegans* embryos elongates more slowly than that in *Xenopus* after MBT. Thus, the T–V relationship in *C. elegans* may be determined by mechanisms different from those used by *Xenopus* embryos. Alternatively, there remains a possibility that a same mechanism functions for the time-size relationship in *C. elegans* and *Xenopus* embryos. In *Xenopus* embryos, the time-size relationship has only been examined in cells near the animal cap. It is possible that the diversity according to cell lineages is also observed in the *Xenopus* embryo. Recently, it has been reported that nuclear size correlates non-linearly with cell size in *Xenopus* embryos (Jevtic and Levy, 2015), similarly to *C. elegans* (Figure 5A). In budding and fission yeasts, the relationship between the nuclear and cell volumes was reported to be linear (Jorgensen et al., 2007; Neumann and Nurse, 2007). In addition, nuclear size has been strongly correlated with cell size (Jorgensen et al., 2007; Neumann and Nurse, 2007), even when cell size was changed 35-fold or nuclear DNA content was changed 16-fold in fission yeast (Neumann and Nurse, 2007). The volume ratio of the nucleus to the cell was rapidly corrected by the growth of the cell or nucleus, when the nuclear or cell size was changed by manipulating the genetic or culture conditions (Neumann and Nurse, 2007). Thus, there is a mechanism that links the sizes of the nucleus and cell in yeasts. Although the mechanism to link the sizes of the nucleus and cell in yeasts is different from animal embryos, the interesting correlation of volume ratio between the nucleus and cell in yeasts and animal embryos raise a possibility that the volume ratio can be a general mean by which cells “sense” their size. The power of the time-size relationship was different between *C. elegans* and *Xenopus* embryos, while the time-size relationship may strongly correlate with the volume ratio between the nucleus and cell in *Xenopus* embryos, like *C. elegans* embryos (See Section The Relationship Between the Nuclear and Cell Volumes in the Size-Correlated Classes). As a future issue, it is interesting to test this possibility to seek a general mechanism that coordinates cell size and cell cycle duration in animal embryos.

In *C. elegans*, cell cycle elongation was due to lengthening of the intermitotic, but not the mitotic, phase in size-correlated AB, MS, C, and P lineages. The *C. elegans* embryonic cell cycle is occupied with S phase at least until the 16-cell stage (Edgar and McGhee, 1988). The gradual elongation of cell cycle duration in the size-correlated AB, MS, C, and P lineages was not due to introduction of the Gap phase, rather due to the elongation of the S phase at least before the 16-cell stage. On the other hand, the Gap phase introduction, which first occurs in the daughters of the E cell (Edgar and McGhee, 1988) was accompanied with abrupt deviation from the T–V relationship that followed by cells in the AB and MS lineages (Figures 2B,G). In *Xenopus*, the transition to size-correlated elongation of cell cycle duration is accompanied, in order, by elongation of the S phase just after MBT, introduction of G1 and G2 phases, and elongation of S and G1 phases (Iwao et al., 2005). Durations of the G2 phase are not correlated with cell cycle elongation. Therefore, size-correlated elongation of the cell cycle duration in *C. elegans* embryos is caused by a cell cycle control mechanism different from *Xenopus*.

### Possible model to explain the power law relationship between cell cycle duration and cell volume in the highly size-correlated class

The *C. elegans* cell cycle duration is likely elongated by S phase elongation at least before the 16-cell stage (Edgar and McGhee, 1988). DNA replication is initiated from specific sites in the chromosomes in eukaryotic cells, called replication origins (Costa et al., 2013). The initiation step of DNA replication, or origin firing, is tightly controlled by the interaction of the replication origin with the initiation factors for DNA replication (IFs) to ensure that the entire genome is replicated precisely once in each cell cycle (Pospiech et al., 2010; Costa et al., 2013). It has been reported that IFs were involved in the asynchrony of division timing of AB and P1 cells (Benkemoun et al., 2014). The asynchrony was explained by the different frequency of the origin firing, such that the length of DNA replication responsible for a single origin may be different between AB and P1 cells (Benkemoun et al., 2014). In this explanation, more origin firing in AB, with DNA replication proceeding from more origins, a shorter time is needed to completely replicate the whole genome DNA than less origin firing in P1.

In *C. elegans* embryos depleted of the ataxia telangiectasia mutated (ATM)-like kinase, *atl-1*, and the checkpoint kinase, *chk-1*, the AB and P1 cell divisions are more synchronous than in wild-type embryos (Brauchle et al., 2003). In *Xenopus* egg extract, ATM-related (ATR)/Chk1 signaling regulates the initiation and progression of DNA synthesis in S phase, even in the absence of DNA damage (Marheineke and Hyrien, 2004; Shechter et al., 2004), probably through modulating the activity of S phase-promoting kinases (Cdk2 and Cdc7) (Marheineke and Hyrien, 2004; Shechter et al., 2004). Therefore, through the regulation of S phase-promoting kinases, ATR/Chk1 signaling may eventually regulate the rate of replication origin firing. Although it remains unknown whether IFs and ATR/Chk1 signaling affect cell cycle duration in *C. elegans* embryos after the two-cell stage, the elongation of cell cycle duration observed in this work may be regulated by the differential regulation of origin firing rate.

We found that the cell cycle was rapidly elongated in *ima-3* RNAi embryos. It has been reported that loss-of-function embryos of *ima-3* exhibit an embryonic lethal phenotype (Geles and Adam, 2001). The rapid elongation of cell cycle in *ima-3* RNAi embryos may be caused by pleiotropic effects of the embryonic lethal phenotype in the late embryonic stage. However, the cell cycle elongation in *ima-3* RNAi embryos was gradual, but not stepwise, such that the slope can be fitted by a power law function. One interesting possibility is that *ima-3* is directly involved in a mechanism regulating the cell cycle progression in *C. elegans* embryos, especially under the control of cell size. The *importin* family encodes proteins that mediate nuclear import and various molecular processes, including transcription by RNA polymerase III, spindle formation, chromosome segregation, and nuclear envelope assembly (Adam, 2009). Further analyses are necessary to study which of IMA-3 functions causes the rapid elongation of cell cycle in *ima-3* RNAi embryos. Importin α was found to be involved in nuclear size determination (Levy and Heald, 2010; Edens et al., 2013). Recently, it has been shown that by manipulating expression or function of factors to regulate nuclear size, including Importin α, it has been shown nuclear size contributes to the regulation of MBT timing in *Xenopus* embryos (Jevtic and Levy, 2015). The same mechanism could function in the T–V relationship in *C. elegans* embryos. Although it remains an open question whether loss of function of *ima-3* leads to altering of nuclear volume in *C. elegans*, there are two intriguing possibilities that IMA-3 mediates the T–V relationship; (1) by regulating nuclear import rate of IFs and/or (2) by regulating nuclear size. Both of the mechanisms affect the T–V relationship through determining their nucleoplasmic concentration of IFs. It will be necessary to test whether one or both of these possibilities were true for evaluating our hypothesis. A theoretical formularization was discussed based on the results of quantitative measurements in this work (Appendix).

### Conflict of interest statement

The authors declare that the research was conducted in the absence of any commercial or financial relationships that could be construed as a potential conflict of interest.

## Supplementary material

Figure S1 **Integral approach for measuring cell volume. (A)** Serial images of an embryo at the four-cell stage along the Z-axis at 3-μm intervals. The image at 0 μm was at the closest side of the embryo to the objective lens. The focus position moved to the farther side of the embryo in an upright microscope. Cell contours were shown as blue circles, which were traced by following the cell periphery and granules in focus located around the cell boundary. **(B)** Three-dimensional reconstruction of an embryo in the two-cell stage from serial images obtained along the Z-axis at 0.5-μm intervals using Image J. In the reconstructed image in the X-Z axis, nuclear peripheries are indicated by black dots.

Figure S2 **Statistical analyses to select a model to explain the T–V relationships in *C. elegans***. Relationship between cell cycle duration and cell volume in AB **(A)**, MS **(B)**, C **(C)**, P **(D)**, E **(E)**, and D **(F)** lineages in the linear scale was fitted by three different models: Gaussian, exponential, or power law function by the Levenberg-Marquardt algorithm, using the same data used in Figure 2. Fitted functions and χ^2^-values are shown in the boxes.

Figure S3 **Size asymmetry between daughter cells in *C. elegans* C lineage**. Diagram shows the cell division pattern in C lineage. Anterior and posterior daughters are indicated as “a” and “p,” respectively. The cell sizes were observed in serial images along the Z-axis, and are shown at the Z axis plane, where the nuclei are located in focus with blue circles. Cell division of Ca, Caa, Cp, and Cpa was asymmetric in the daughter cell sizes, in which the anterior daughter was larger than the posterior daughter. Cpa and Cpp were located in different Z-planes in an embryo. Scale bar = 10 μm.

## Appendix

### Numerical formularization of our model

We showed that the *C. elegans* T–V relationship in the highly size-correlated class correlated with the volume ratio between the nucleus and cell (Section The Relationship Between the Nuclear and Cell Volumes in the Size-Correlated Classes). Here, we discuss a possible molecular mechanism to regulate the T–V relationship (Section Possible Model to Explain the Power Law Relationship Between Cell Cycle Duration and Cell Volume in the Highly Size-Correlated Class), based on a theoretical numerical formularization of our model. The causal relationship between the cell cycle duration and cell volume has not previously been shown in *C. elegans* embryos, and knowledge of molecular mechanisms regulating cell cycle duration are insufficient. Thus, this model does not exclude alternative numerical models.

We presume that the cell cycle duration in the highly size-correlated class (*T*), which is occupied with S phase, is determined by the whole genome size (*L*; [n.a.]), the replication velocity (*k*, [n.a./s]) which is assumed to be constant in embryonic cells, and the number of replication origins that are fired during S phase (*N* [−]), where. n.a. indicates the number of nucleic acids:

(A1) $$T=\frac{L}{N\cdot k}$$

In addition, we presumed that the number of fired origins during S phase is in proportion to the concentration of the IFs in the nucleoplasm ([*nucIF*]):

(A2) N∝[nucIF]

We also presumed that the total amount of IFs is constant in embryos. The amount of IFs in each cell (*cyto*IF) is determined following the segregation of daughter cells from the mother cell according to the daughter cell volume. The nucleoplasmic IF concentration is presumed to be determined by active transport via the Importin system (Section Possible Model to Explain the Power Law Relationship Between Cell Cycle Duration and Cell Volume in the Highly Size-Correlated Class), so that the nucleoplasmic concentration of the IFs is reduced according to embryonic cell cleavage events that follow the power law of the volume ratio between the nucleus and cell (Figure 5B). Consequently, the T–V relationship in the highly size-correlated class elongated according to the volume ratio between the nucleus and cell.

(A3) $$\left[nucIF\right]\propto \frac{cytoIF}{V_{n}}\propto \frac{V_{c}}{V_{n}}$$

From Equations (A2, A3):

(A4) $$N\propto V_{c}/V_{n}$$

From Equations (A1, A4):

(A5) $$T\propto \frac{L}{V_{c}/V_{n}\cdot k}\propto \frac{V_{n}}{V_{c}}$$

From our experimental measurements of nuclear and cell volume (Section The Relationship Between the Nuclear and Cell Volumes in the Size-Correlated Classes),

(A6) $$\frac{V_{n}}{V_{c}}\propto V_{c}^{-0.37}$$

Therefore, from Equations (A5, A6):

$$T\propto V_{c}^{-0.37}.$$

Thus, the cell cycle duration in the highly size-correlated class can be determined by the amount of IFs that are inherited in proportion to cell size and function in proportion to nuclear size. Eventually, cell cycle duration is elongated in proportion to cell volume with the power −0.37.
