# Supplementary figures and images for: Power law relationship between cell cycle duration and cell volume in the early embryonic development of Caenorhabditis elegans
Source: Front Physiol. 2015 Jan 28;5:529. doi: 10.3389/fphys.2014.00529 (PMC4309120; doi:10.3389/fphys.2014.00529)

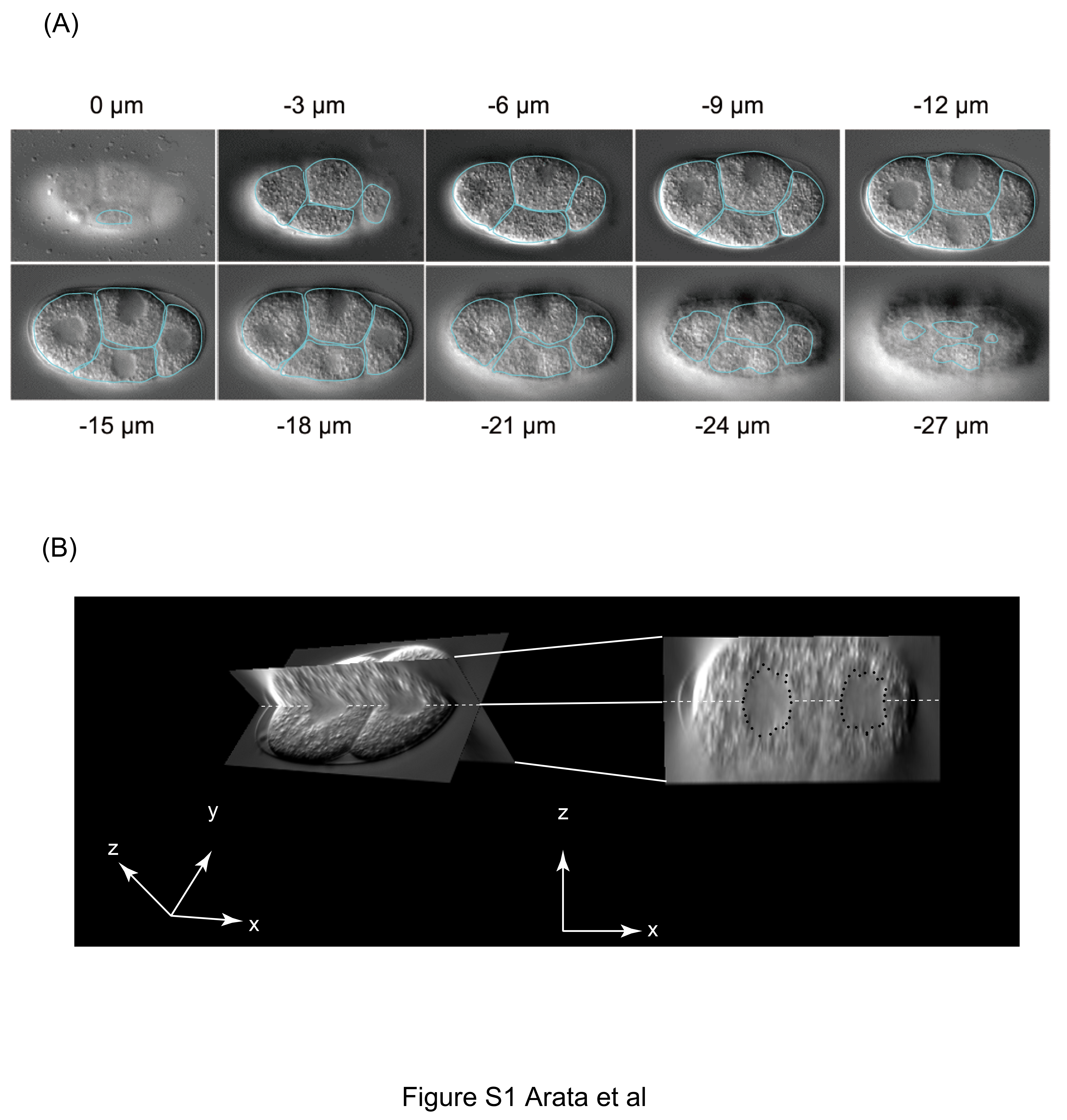

Supplement: Figure S1 — Integral approach for measuring cell volume. (A) Serial images of an embryo at the four-cell stage along the Z-axis at 3-μm intervals. The image at 0 μm was at the closest side of the embryo to the objective lens. The focus position moved to the farther side of the embryo in an upright microscope. Cell contours were shown as blue circles, which were traced by following the cell periphery and granules in focus located around the cell boundary. (B) Three-dimensional reconstruction of an embryo in the two-cell stage from serial images obtained along the Z-axis at 0.5-μm intervals using Image J. In the reconstructed image in the X-Z axis, nuclear peripheries are indicated by black dots. [file Image1.TIF]

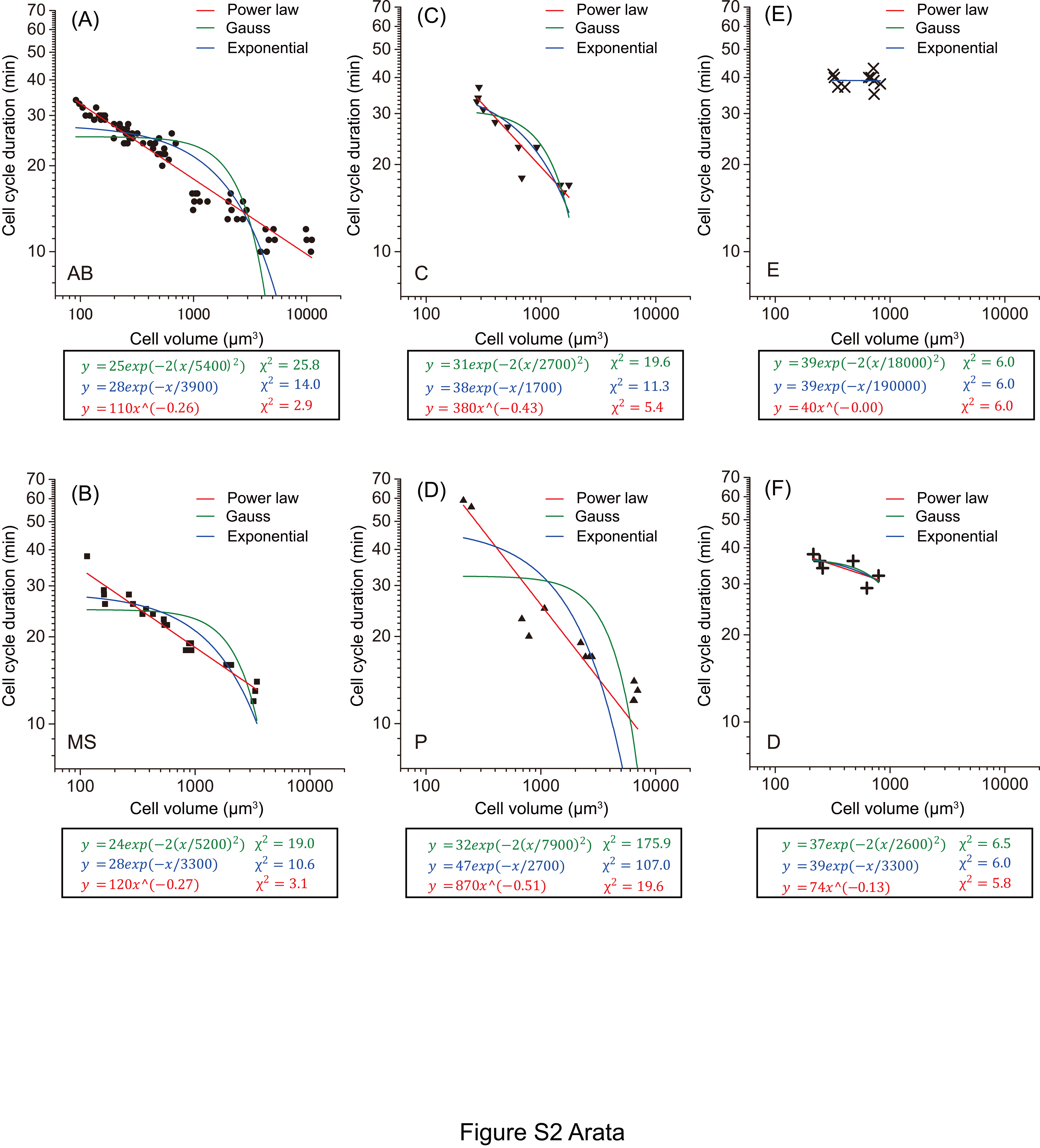

Supplement: Figure S2 — Statistical analyses to select a model to explain the T–V relationships in C. elegans. Relationship between cell cycle duration and cell volume in AB (A), MS (B), C (C), P (D), E (E), and D (F) lineages in the linear scale was fitted by three different models: Gaussian, exponential, or power law function by the Levenberg-Marquardt algorithm, using the same data used in Figure 2. Fitted functions and χ2-values are shown in the boxes. [file Image2.TIF]
